# Cutaneous metastasis of occult breast cancer: a case report

**DOI:** 10.11604/pamj.2021.40.23.31009

**Published:** 2021-09-08

**Authors:** Rafael Everton Assunção Ribeiro da Costa, Cristiane Amaral dos Reis, Rafael de Deus Moura, Ana Lúcia Nascimento Araújo, Fergus Tomás Rocha de Oliveira, Sabas Carlos Vieira

**Affiliations:** 1 State University of Piauí, Teresina, PI, Brazil,; 2 Oncocenter, Teresina, PI, Brazil,; 3 Federal University of Piauí, Teresina, PI, Brazil

**Keywords:** Breast neoplasms, unknown primary, neoplasm metastasis, skin neoplasms, case report

## Abstract

Occult breast cancer (OBC) is characterized by metastatic presentation of undetectable breast tumor on imaging exams. OBC is a rare disease (accounting for 0.3% to 1.0% of all breast cancers) that represents a major diagnostic challenge. The aim of this study was to report a case of OBC with primary presentation of multiple cutaneous metastases with subsequent emergence of bone metastasis. A 70-year female patient had multiple cutaneous metastatic lesions in the left cervical region, left breast, left axillary region, left subscapular region, in three chirodactylus of the right hand and three chirodactylus of the left hand. Imaging tests (mammogram, ultrasonography and magnetic resonance imaging of the breast) did not show alterations. Biopsy, histology sections and immunohistochemistry of the left cervical cutaneous lesion were compatible with OBC. After two years of anastrozole treatment (1mg/day), there was regression of all cutaneous lesions and stabilization of bone metastasis. OBC has a better prognosis. It may exhibit spontaneous regression or respond to less aggressive treatment strategies, as described in this case.

## Introduction

Occult breast cancer (OBC) is defined as the clinical presentation of metastatic carcinoma (mainly in axillary lymph nodes) derived from a malignant primary breast tumor that is undetectable by clinical exams and radiological evaluation. OBC is a rare condition, accounting for 0.3%-1.0% of all breast cancers. It occurs most commonly at around 55 years of age [1, 2]. Cutaneous metastases may be considered rare dermatological manifestations that occur in around 0.7% to 0.9% of cancer patients. Therefore, advanced breast cancer is implied in the occurrence of cutaneous metastases in cancers in general [3]. OBC represents a diagnostic challenge and is a rare event, especially with primary manifestations of systemic metastasis. Therefore, the aim of this study was to report a case of OBC, mainly manifested as cutaneous metastases and subsequent detection of bone metastasis.

## Patient and observation

**Patient information:** a 70-year old female patient, G1P0A0, non-smoker, non-alcoholic, hypertensive and sedentary.

**Figure 1:**
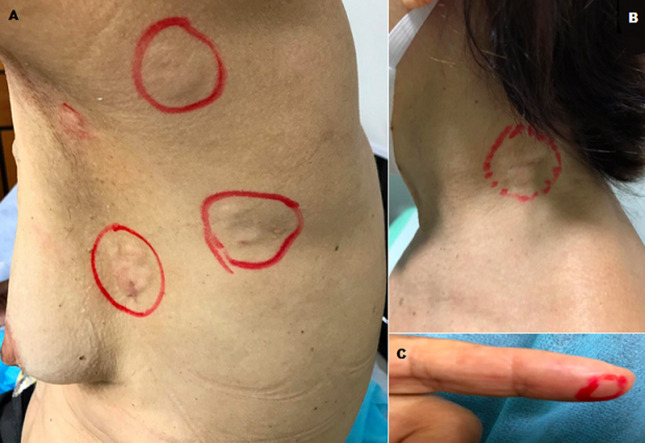
cutaneous metastases of occult breast cancer in the left breast, left axillary region and left subescapular region (A), left cervical region (B) and second left chirodactylus of the patient (C)

**Clinical findings:** the patient presented multiple cutaneous metastatic lesions in the left breast, left axillary region and left subscapular region (Figure 1A), left cervical region (Figure 1B), as well as in three chirodactylus of the right hand and three chirodactylus on the left hand (Figure 1C, an example in the second left chirodactylus).

**Timeline of current episode:** August 2018: biopsy, histology and immunohistochemical study were conducted. March 2019: patient referral to the healthcare unit. October 2020: bone scintigraphy was performed. February 2021: a new bone scintigraphy was performed. March 2021: disappearance of all metastatic lesions of the OBC.

**Figure 2:**
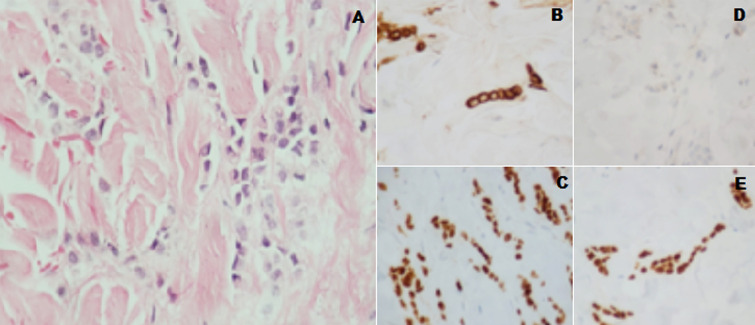
(A) histopathology (hematoxylin-Eosin staining), (B) pan cytokeratin, (C): ER, (D): HER2 and (E): GATA-3; all slides at x400 magnification

**Diagnostic assessment:** biopsy, histology sections and immunohistochemistry of skin lesion in the left cervical region (Figure 2) indicated: grade 2 or moderately differentiated lobular carcinoma, pan cytokeratin positive, cytokeratin 7 (CK7) positive, estrogen receptor (ER) positive, progesterone receptor (PR) positive, human epidermal growth factor 2 (HER2) negative and GATA 3-protein gene (GATA-3) positive. Mammography, ultrasonography and magnetic resonance imaging of the breast were performed. No structural alterations were found in any of these exams. Chest and abdominal CT-scans did not demonstrate metastasis. The patient did not perform PET-CT due to unavailability of the exam. A bone scintigraphy showed areas of increased uptake in the 8th and 9th right costal arcs that were suspected of metastatic disease. A new scintigraphy indicated stabilization of the bone uptake detected.

**Diagnosis:** the results were consistent with breast cancer metastases. A diagnosis of lobular breast carcinoma of unrecognized primary site (OBC) with cutaneous metastatic dissemination was made. The first bone scintigraphy showed areas of increased uptake in the 8^th^ and 9^th^ right costal arcs suspicious for metastatic disease.

**Therapeutic interventions:** anastrozole was initiated (1mg/day).

**Follow-up and outcome of interventions:** the patient had an excellent response to anastrozole. She remains asymptomatic and in excellent clinical condition.

**Patient perspective:** “I have an expectation of being cured of cancer, given that some conditions have completely disappeared and I continue with treatment and clinical follow-up”.

**Informed consent:** the current study is part of a scientific project that was approved by the research ethics committee (REC) of the State University of Piauí, Teresina, PI, Brazil, under number CAAE: 30154720.0.0000.5209. All ethical principles established by the National Health Council resolution number 466/12 and international documents were followed. The patient gave informed consent.

## Discussion

OBC is a rare and challenging diagnosis. In the literature, there is a paucity of conclusive studies on the clinical-pathological characteristics of the disease, as well as patient outcome and disease management. Huang *et al*. analyzed 572 OBC patients in comparison to 117.217 cases of non-occult breast cancer. Those authors also concluded that OBC patients are diagnosed at a more advanced age, as occurred in the case report [4].

Cutaneous metastases due to breast cancer are uncommon, indicating that internal malignancies occur [3]. The first manifestation of OBC is axillary node involvement. Primary occurrence of distant metastasis is less common [5]. In the patient described, multiple cutaneous metastases were the early manifestations of OBC with subsequent detection of probable bone metastasis. Imaging techniques currently used for diagnosis of breast cancer (mammography, ultrasonography and magnetic resonance imaging) are not sensitive for OBC [6]. Nevertheless, immunohistochemical analysis of nodal or systemic presentations, with the characteristic markers of breast carcinomas, such as CK7, estrogen and progesterone receptors and GATA-3, can determine that the breast is the primary site of cancer [7].

Furthermore, OBC has a better prognosis [4, 8]. In the English literature, a case of spontaneous regression (partial or total disappearance of cancer without any type of treatment) of nodal metastasis in OBC was reported [9]. In this case report, the patient with OBC had a good clinical outcome. Regression of cutaneous metastasis and stability of bone metastasis occurred, and anastrazole alone was used for treatment.

We identified three articles reporting cases of cutaneous metastasis of OBC by using the following search strategy on PubMed: *"skin AND occult AND metastasis AND breast AND cancer"*[3, 10, 11] (Table 1). In the three other cases identified in PubMed, patients had a more aggressive disease than the patient described in this case report, requiring more robust treatment strategies, such as mastectomy, chemotherapy and radiotherapy. Therefore, this case report is quite interesting and rare. In addition, it may be considered unique in relation to the clinical outcome presented.

**Table 1:** description of similar studies found in literature by PubMed search (case reports of patients with cutaneous metastasis of OBC)

Authors	Year	Patient characterization (age at diagnosis and metastatic presentation)	Positive immunohistochemistry results	Treatment and outcome
Alizadeh *et al*. [3]	2018	44-year old woman with cutaneous metastasis in the scalp.	Cytokeratin (AE1/AE3), cytokeratin 7, chromogranin, estrogen, progesterone and HER2 receptors and macroscopic cystic fluid protein 15.	Chemotherapy (4 cycles of adriamycin and cyclophosphamide, followed by 4 cycles of docetaxel). Surgical resection and graft in the scalp. Radiotherapy and tamoxifen 20 mg/day. Patient had a favorable outcome at 3 years of follow-up, without any recurrences.
Weimann *et al*. [10]	2016	65-year woman with cutaneous metastasis in the upper body and upper limbs.	Cytokeratin (AE1/AE3), cytokeratin 7, mammaglobin and estrogen receptors.	Patient was referred to chemotherapy and clinical follow-up, but died soon afterwards (10 months after diagnosis).
Cohen-Kurzrock *et al*. [11]	2021	30-year old woman with cutaneous metastasis in the breasts, axillary region and groin.	Not detailed.	Mastectomy of the right breast and adjuvant chemotherapy. Patient under chemotherapy at the time of case publication.

## Conclusion

OBC has a better prognosis, with possibility of spontaneous tumor regression or response to less aggressive treatment strategies. At two years of follow-up, the patient in this case report had an excellent clinical outcome, with regression of all cutaneous metastatic lesions. Bone metastasis was stable with the sole use of anastrozole (1mg/day) for treatment.
